# Workup and Management of Recurrent Attacks of Post-bariatric Hypoglycemia in a Patient With Non-alcoholic Steatohepatitis

**DOI:** 10.7759/cureus.39544

**Published:** 2023-05-26

**Authors:** Sindhu C Pokhriyal, Sagar Nagpal, Uma Gupta, Parjanya K Bhatt, Pulok Roy, Nway Nway, Sunil Parkash, Ruchi Yadav, Zewge Shiferaw-Deribe

**Affiliations:** 1 Internal Medicine, One Brooklyn Health, New York, USA; 2 Department of Internal Medicine, University at Buffalo, Buffalo, USA; 3 Internal Medicine, Interfaith Medical Center, New York, USA; 4 Department of Medicine, Byramjee Jeejeebhoy (BJ) Medical College, Ahmedabad, IND; 5 Hematology and Oncology, Brookdale University Hospital Medical Center, Brooklyn, USA; 6 Internal Medicine/Endocrinology, Interfaith Medical Center, Brooklyn, USA

**Keywords:** hypoglycemia workup, nocturnal episodes, gastric bypass surgery, nonalcoholic steatohepatitis, post-bariatric hypoglycemia

## Abstract

Bariatric surgery is an established treatment option for patients with non-alcoholic fatty liver disease (NAFLD) as well as non-alcoholic steatohepatitis (NASH) and is said to effectively reduce hepatic inflammation as well as steatosis in these patients. However, bariatric surgery is associated with multiple complications, including nutritional deficiencies, malnutrition, post-bariatric hypoglycemia (PBH), anastomotic leaks, and bowel strictures. This case report describes a rare but significant complication of post-bariatric surgery hypoglycemia in a patient with NASH, which started almost six months after Roux-en-Y gastric bypass (RYGB) surgery. This 55-year-old male patient presented with recurrent episodes of severe hypoglycemia, which, on further work-up, were found to be predominantly nocturnal as well as occurring two to three hours after meals. We report the successful treatment of the patient with an unconventional approach using nifedipine and acarbose. Our findings emphasize the importance of careful evaluation of patients who have undergone bariatric surgery, as this complication can occur as early as six months following the bariatric surgery as well as several years after the surgery. Our case report highlights the need for early recognition, relevant workup, and appropriate management of resistant hypoglycemic events using calcium channel blockers and acarbose, thus adding to the existing literature on this topic.

## Introduction

Non-alcoholic fatty liver disease (NAFLD) is defined as a substantial buildup of triglycerides in hepatocytes, as determined by imaging or histology, in the absence of significant alcohol consumption or other liver diseases. When NAFLD is associated with hepatic inflammation and hepatocellular steatosis, it is said to have progressed to non-alcoholic steatohepatitis (NASH). Bariatric surgery is one of the most effective treatments for long-term weight loss as well as reversing inflammatory and fibrotic changes in the liver secondary to steatohepatitis in patients with NAFLD/NASH [1]. Some types of bariatric surgery include adjustable gastric bands, sleeve gastrectomy, and Roux-en-Y gastric bypass (RYGB) surgery. Overall, cases of bariatric surgery are increasing every year, and the field is expected to continue to grow with increasing obesity [2]. There are many well-known complications of bariatric surgery, and post-bariatric hypoglycemia (PBH) is one of them. The prevalence of PBH is approximately between 0.2% and 0.36%, based on a large cohort study [3,4]. The diagnosis is usually based on history, followed by an evaluation to check if Whipple’s triad is met as well as establish the pattern of hypoglycemia [5]. PBH typically usually occurs one to three hours after meals, usually after intake of high-glycemic-index foods, exhibiting a classic delayed postprandial pattern [5]. Nocturnal episodes of hypoglycemia are an uncommon presentation of PBH. PBH as a condition is associated with severe morbidity and has several socioeconomic implications for the patient's life [6]. Treatment options include nutritional modifications, medical therapies like acarbose, nifedipine, octreotide, diazoxide, GLP-1 agonists (glucagon-like peptide-1), and surgical options [6,7]. Here, we present a case of post-bariatric hypoglycemia with post-meal as well as nocturnal hypoglycemic episodes in a patient with background NASH and diabetes that was successfully treated along with medical therapy.

## Case presentation

A 55-year-old male presented to the emergency department with lethargy and confusion due to hypoglycemia, as the point-of-care glucose testing showed glucose levels of 28 mg/dL, which responded well to glucagon. His past medical history was relevant for insulin-dependent type 2 diabetes mellitus, hypertension, hyperlipidemia, robot-assisted laparoscopic gastric bypass six months ago, and liver cirrhosis secondary to NASH. His liver disease had been diagnosed two years before presentation by liver biopsy while he was being worked up for elevated liver enzymes. He was prescribed metoprolol, spironolactone, and furosemide for the management of liver cirrhosis with ascites secondary to NASH. All of his diabetes medications, including insulin, had been gradually tapered off over the past few months due to consistently decreasing blood glucose levels. He had been entirely off insulin for over two months at the time of presentation. His basal metabolic index (BMI) at the time of the bariatric surgery was 39, and he reported a weight loss of more than 100 pounds in the last six months, which was nearly 30% of his total excessive weight. Physical examination upon presentation revealed a notable BMI of 19 and evident clinical ascites. The metoprolol was withheld due to hypoglycemia unawareness, and dextrose-based IV fluids were commenced. The patient persistently experienced recurrent episodes of nocturnal hypoglycemia, even in the presence of dextrose-based infusions. The comprehensive workup for hypoglycemia included insulin, pro-insulin, C-peptide, a sulfonylurea screen, and beta-hydroxybutyrate levels and revealed several notable findings. The C-peptide level was measured at 0.6 ng/mL (reference range 0.5 to 2 ng/mL), indicating low levels. Proinsulin was within the normal range at 1.5 pmol/L (3-20 pmol/L), and insulin levels were also normal at 8.3 mU/L (2.6-24 mU/L). The fasting beta-hydroxybutyrate level was 0.2 mmol/L (reference range 0.4-0.5 mmol/L), slightly below the normal range. Additionally, the insulin-to-C-peptide molar ratio was less than 1, ruling out the possibility of an insulinoma. The sulfonylurea screen yielded negative results. Glycosylated hemoglobin was measured at 6.4% (reference range: <6.5%), indicating good glycemic control. Insulin antibody levels were positive, with slightly elevated values of 7.6 µU/mL (reference range: <5 µU/mL). It was noted that antibodies to exogenously administered insulin are common in patients on insulin therapy, but they are frequently clinically insignificant and rarely might suggest insulin resistance. Furthermore, the insulin-like growth factor-1 level was found to be low at 33 ng/mL (reference range 74-255 ng/mL), likely due to the presence of cirrhosis. Other lab tests were unremarkable, as shown in Table 1.

**Table 1:** Patient laboratory findings in the current admission.

Laboratory test	Normal range	Results
White blood cell	4.5–11.0 × 10^3^/µL	5.2
Hemoglobin	11.0–15.0 g/dL	11.5
Platelets	130–400 × 10^3^/µL	388
Blood urea nitrogen	9.8–20.1 mg/dL	15
Creatinine	0.57–3.1.11 mg/dL	0.9
Potassium	3.5–5.1 mmol/L	5
Sodium	133–145 meQ/L	137
Total bilirubin	0.2–1.3 mg/dL	0.6
Alanine transaminase	17–63 U/L	21
Aspartate transaminase	0–37 U/L	25
Albumin	3–5.5 g/dL	2.7
Lactic acid	0.7–2.1 mmol/L	1.5
Prothrombin time	9.8–13.4 seconds	13.2
International normalized ratio	0.85–1.15 ratio	1.02
High sensitivity troponin	5–17 ng/mL	<5, <5
Partial thromboplastin time	24.9–35.9 seconds	36
Thyroid-stimulating hormone	0.465–4.680 µIU/mL	3.1
Free thyroxine (T4)	0.78–2.19 ng/dL	1.66
Brain natriuretic peptide	10.0–100 pg/mL	94
Ammonia	9–30 µmol/L	32
Copper	69–132 µmol/dL	56
Anti-smooth muscle antibody	0–19 units	10
Anti-mitochondrial antibody	0–20 units	<20
Antinuclear antibody	<1:80	Negative
Liver kidney microsomal antibody	0–20 units	<20

**Figure 1:**
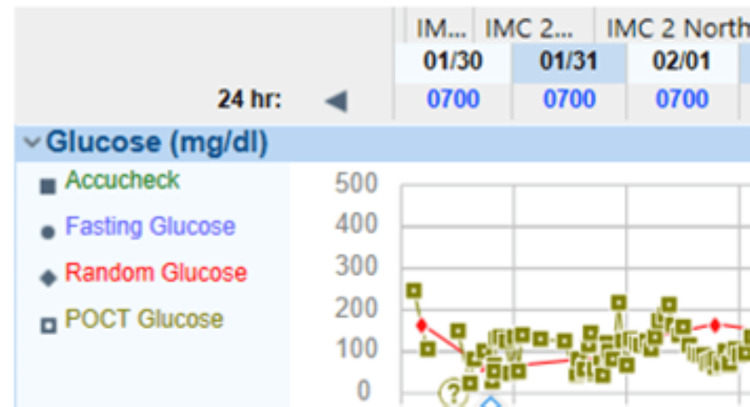
Graphical representation of hypoglycemic episodes on hourly monitoring.

A mixed meal test was scheduled; however, it could not be completed due to the patient's inability to tolerate it. The patient was placed on continuous glucose monitoring to establish the pattern of hypoglycemia, especially in relation to meals. The glycemic pattern was characteristic of hypoglycemic episodes two to three hours post meals and predominant nocturnal hypoglycemia (Figure 1).

**Figure 2:**
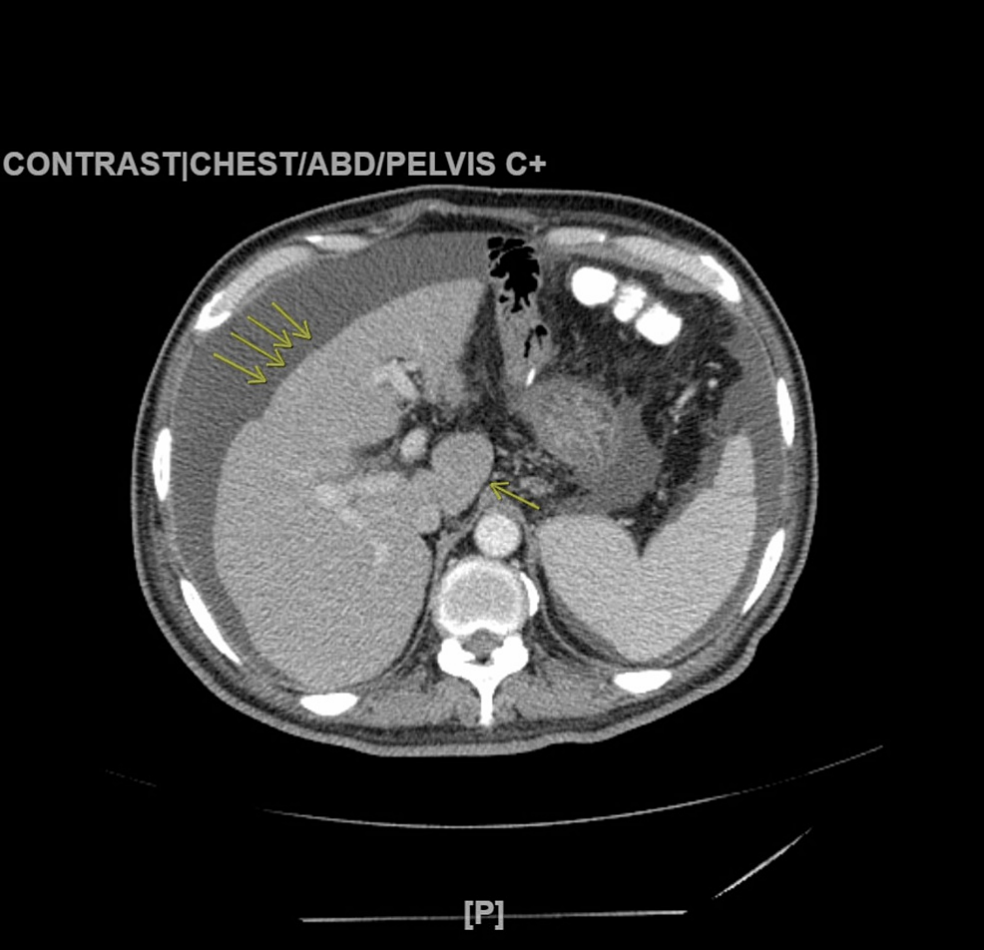
CT abdomen with multiple arrows showing the nodular shrunken and cirrhotic liver; the single arrow on the right pointing to the enlarged caudate lobe.

**Figure 3:**
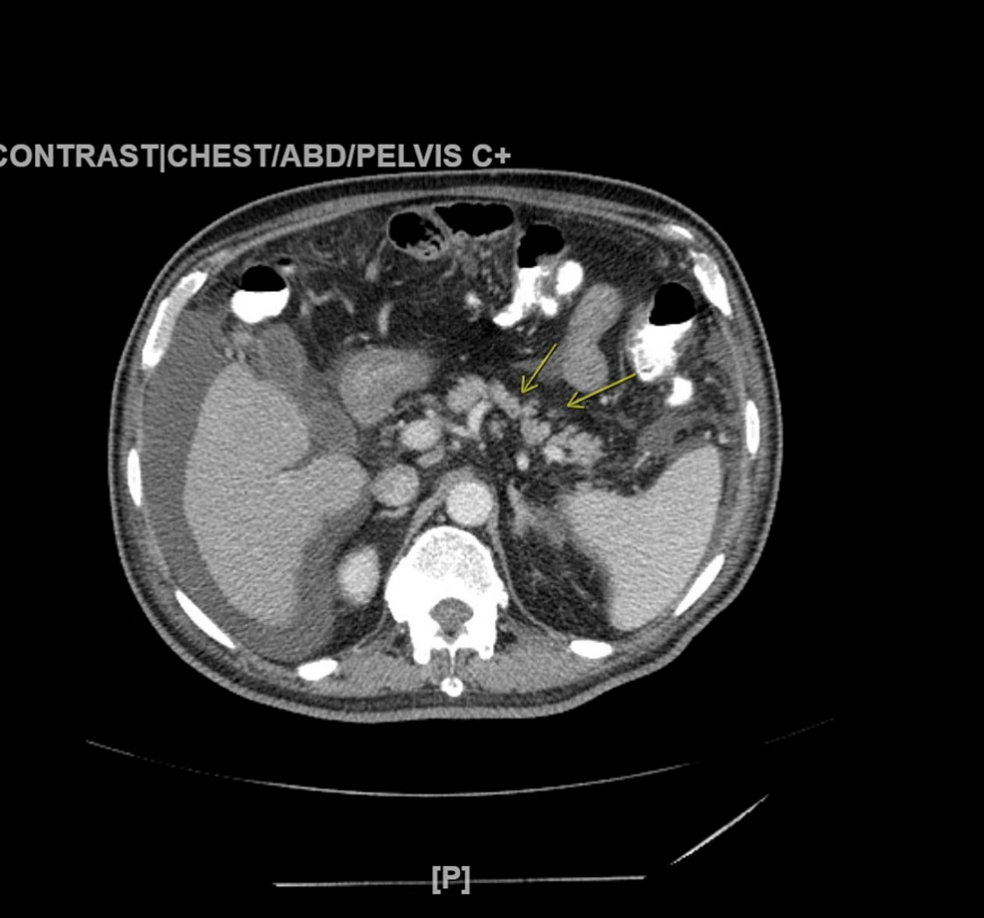
Partial fatty infiltration of the pancreas.

A cosyntropin test was performed and yielded negative results, ruling out adrenal insufficiency. Abdominal computed tomography revealed partial fatty infiltration of the pancreas, cirrhotic changes in the liver, including the enlarged caudate lobe, and the presence of ascites (as shown in Figures 2-3).

**Figure 4:**
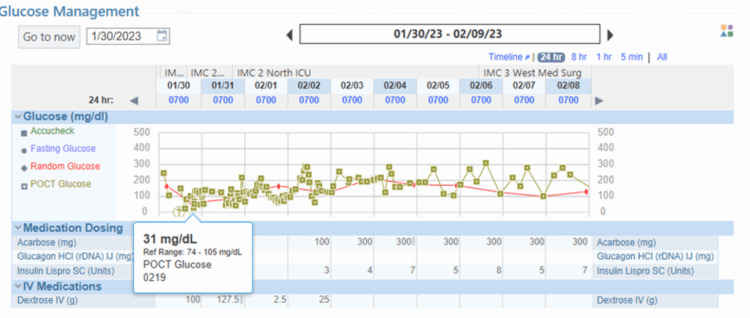
Graphical representation of resolution of hypoglycemic episodes after starting acarbose and nifedipine.

Ascitic fluid cytology showed no evidence of malignancy, and fluid analysis confirmed it to be transudate. The serum ascites albumin gradient of >1.1 g/dL suggested portal hypertension secondary to cirrhosis. For severe hypoglycemic episodes with blood glucose levels below 40 mg/dL, the patient received dextrose 50% and glucagon. Due to persistent nocturnal hypoglycemia and impaired awareness of hypoglycemia, the patient was transferred to the intensive care unit for closer monitoring. In addition to managing hypertension, nifedipine 60 mg daily was initiated to address possible post-bariatric hypoglycemia. The blood pressure was well controlled with nifedipine. The frequency of hypoglycemic episodes was reduced but did not completely resolve after starting nifedipine. Acarbose, 100 mg three times daily, was subsequently added to the treatment regimen. The patient responded well to the combination of acarbose and nifedipine, leading to the resolution of both daytime and nocturnal episodes of hypoglycemia (Figure 4). On the fifth day of admission, the patient was discharged in stable condition. On a follow-up visit after a month, the patient was doing well and was counselled to maintain compliance with lifestyle and medications.

## Discussion

**Figure 5:**
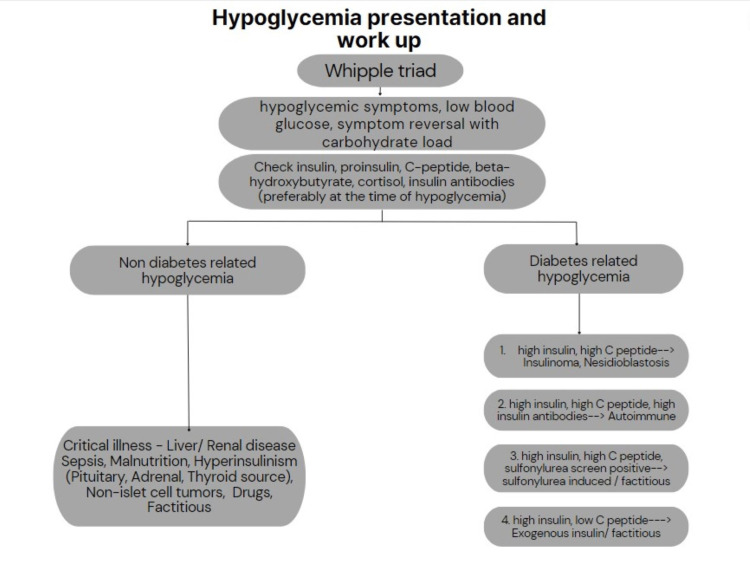
Flowchart describing the diagnostic work-up of a patient presenting with hypoglycemia.

It is widely recognized that the diagnosis of hypoglycemia is established using the classic Whipple's triad. Whipple's triad encompasses episodic hypoglycemia, neuroglycopenic symptoms, and the prompt resolution of these symptoms upon administration of glucose [5]. Hypoglycemia can occur in the presence of various comorbidities and critical conditions, including liver disease, kidney disease, sepsis, cardiac failure, and Addison's disease [5]. Furthermore, several medications, such as quinolones, pentamidine, angiotensin-converting enzyme inhibitors, and beta blockers, can induce hypoglycemia. Medication-induced hypoglycemia is particularly prevalent in elderly individuals with other comorbidities like liver failure or renal failure, as depicted in Figure 5 [6,8].

Non-diabetic hypoglycemia in adults is much rarer than diabetic hypoglycemia. Our patient had diabetes and had been free from all hypoglycemic medications for over two months, indicating a period of remission. However, he presented to us with recurrent episodes of level 3 hypoglycemia, with blood glucose levels falling below 55 mg/dL, accompanied by neuroglycopenic symptoms.

Our patient had not been on any of the hypoglycemia-inducing medications. However, he had NAFLD and subsequently developed NASH. It is well recognized that bariatric surgery is recommended in patients with NAFLD and NASH, as it is known to decrease and reverse the histological features of NASH like inflammation and fibrosis. Bariatric surgery is considered superior to non-surgical treatment modalities like diet and exercise in the management of obese patients with NASH [1,9]. However, unfortunately, bariatric surgery, like all surgical interventions, is associated with certain debilitating complications, and PBH is one such complication.

The incidence of PBH was 0.2% among all patients who underwent BS [3,4]. PBH commonly presents as postprandial hypoglycemia within one to three hours of meal intake. Nocturnal hypoglycemia is an uncommon manifestation of PBH [5]. A cross-sectional study in patients with Roux-en-Y gastric bypass (RYGB) and sleeve gastrectomy by Lupoli et al. studied the pattern of hypoglycemic episodes in these patients and concluded that patients with gastric bypass have a higher risk of hypoglycemic episodes. However, in their study, they also found that nocturnal hypoglycemic episodes are more common in sleeve gastrectomy when compared to gastric bypass [10]. The pathophysiology of nocturnal hypoglycemia is not fully understood yet. A study by Lee et al. suggested different mechanisms to explain the nocturnal phenomenon. These include delayed effects of post-meal insulin secretion, reduced glycogenolysis either due to reduced glycogen content or resistance to stress hormones, failure of transition to gluconeogenesis, or insulin-independent glucose uptake [6,11]. Larger studies will be required to explain and integrate these possibilities.

Among all bariatric procedures, RYGB is most commonly associated with complications of PBH. This is because it causes some critical changes in glucose metabolism secondary to changes in gut anatomy and weight loss [7]. PBH can lead to life-threatening neuroglycopenic symptoms such as seizures, disorientation, and loss of consciousness, with or without warning signs [4,8]. PBH classically presents one to three years after surgery with features of hypoglycemia such as sweating, tremor, hunger, palpitations, dizziness, bizarre dreams, morning headaches (indicating nocturnal hypoglycemia), and mood swings.

**Figure 6:**
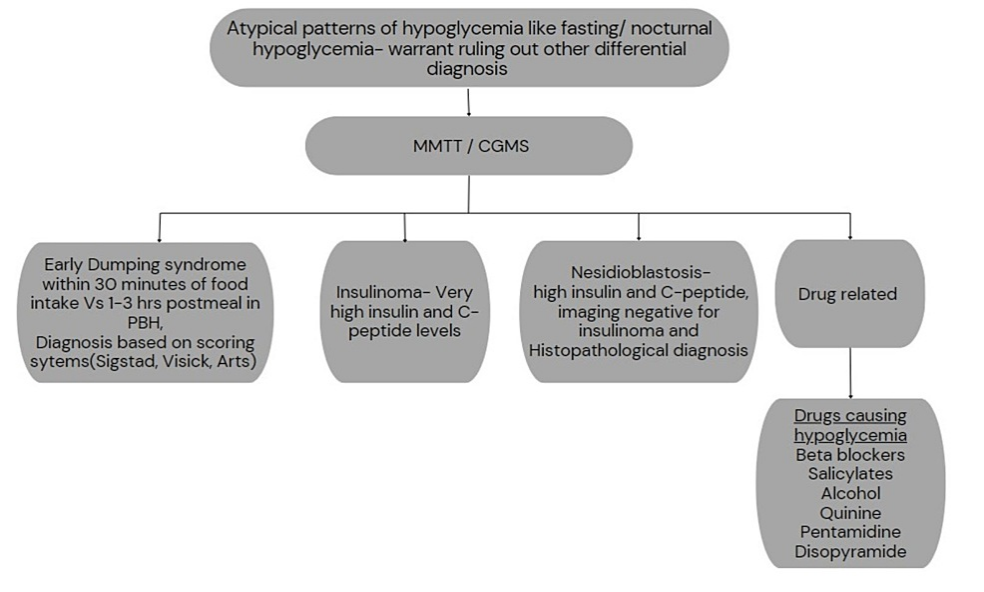
Further workup of atypical patterns of hypoglycemia and the possible causes.

PBH rarely manifests until more than a year after bariatric surgery [5]. Symptomatic hypoglycemia in the fasted state or more than four hours after caloric intake is not typical and should raise concern for other causes of hypoglycemia in the early postoperative period (6 to 12 months). Patients may also report hypoglycemia during periods of increased physical activity or late at night, but these are atypical presentations that must prompt further workup in order to rule out other causes of hypoglycemia, as shown in Figure 6 [6,11].

In critically ill patients, spontaneous episodes of severe hypoglycemia are rare, with an incidence of less than 1.5% of patients, and usually seen in fulminant hepatic failure and/or overt adrenal failure during septic shock, particularly in patients with chronic liver disease, chronic renal failure, or in those with malnutrition [12]. Our patient had cirrhosis with features of portal hypertension like ascites, but he did not have fulminant hepatic failure or adrenal insufficiency. So this seemed less likely to be the cause of the hypoglycemic episodes. However, the exaggeration of PBH symptoms by liver disease cannot be completely ruled out in our patients.

**Figure 7:**
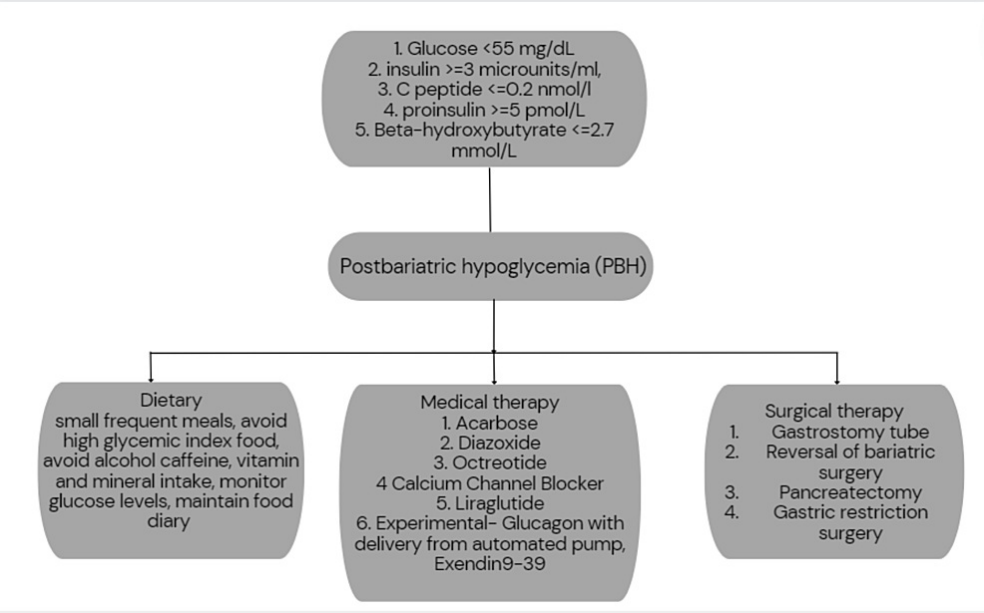
Diagnosis and management of post-bariatric hypoglycemia.

After ruling out other causes of hypoglycemia in patients with a history of bariatric surgery, mixed meal tolerance tests (MMTT) after an overnight fast can be used to diagnose PBH. Glucose levels <55 mg/dl, insulin levels ≥3 µU/ml, C-peptide ≥0.2 nmol/l, and proinsulin ≥5 pmol/l, with suppressed levels of beta-hydroxybutyrate ≤2.7 mmol/l, support the diagnosis of PBH [6]. Ambulatory glucose monitoring using the continuous glucose monitoring system (CGMS) is an alternative when patients cannot undergo MMTT. This helps in identifying the relationship between hypoglycemic symptoms and glucose levels, the frequency, the severity, and the associated pattern. By using CGMS, patients can anticipate and prevent hypoglycemia through changes in diet and self-treatment. Thus, PBH patients, especially those who are unaware of hypoglycemia, may benefit from the use of CGMS devices [13]. We attempted to develop an algorithm for the management of PBH based on our understanding and the literature we reviewed in Figure 7.

Our patient suffered from recurrent attacks of nocturnal hypoglycemia. Pathological conditions such as insulinoma were ruled out. Such episodes are not routinely described in PBH. Explaining patterns of nocturnal hypoglycemia in post-bariatric patients is the scope of future studies. As nocturnal episodes are detrimental to health by impacting cardiovascular function and affecting neuronal and cognitive function, there is a need to further enhance the literature on PBH. Our patient responded to nifedipine and acarbose, and the episodes of hypoglycemia had completely resolved before discharge. There is multidisciplinary team involvement in managing the case. One end of the spectrum lies in preventing such episodes in the first place with dietary modification and strict glucose monitoring. For those refractory to dietary modifications, the addition of medical therapy is advised. If, despite all measures, the patient still continues to have uncontrolled or severe, refractory episodes of PBH, then surgical management is advocated. At times, hypoglycemic episodes go unnoticed, and patients are unaware of hypoglycemia. Continuous glucose monitoring using the CGMS machine can mitigate this issue, helping to prevent the development of neuroglycopenic episodes and further worsening. In our patient's case, at follow-up, the patient was doing well with dietary modifications as well as acarbose and nifedipine.

## Conclusions

PBH is a well-known, debilitating complication of bariatric procedures like the RYGB. Medical management with medications like acarbose and nifedipine can significantly improve the patient's condition and, therefore, their quality of life. It should be noted that symptoms may vary over time, even with strict adherence to treatment regimens. Due to the high glycemic variability and unpredictability of hypoglycemia, optimal management of PBH requires a multidisciplinary approach. To mitigate the risk of hypoglycemia in critical care settings, healthcare providers should implement diligent glucose monitoring (including using a CGMS), investigate the underlying causes and patterns of hypoglycemia, thoroughly assess comorbidities and medications for potential interactions, educate patients and their families about the condition, and make necessary adjustments to diet and medications while remaining vigilant for neuroglycopenic symptoms. Hypoglycemia also has a detrimental impact on patient outcomes, healthcare resources, and expenditure, leading to extended hospital stays. Further research is warranted to better understand the long-term consequences of PBH.
